# In the pursuit of competitive advantage in ophthalmology services

**Published:** 2019

**Authors:** Consuela Mădălina Gheorghe

**Affiliations:** * Philologist, Authorized translator

Competition has become a paramount element in all healthcare markets. Consequently, most healthcare organizations are now actively trying to engage in activities that would bring a competitive advantage. Unfortunately, there are challenging obstacles to surpass, as well as heavily institutional constraints, which steam from the extensive array of regulations and ethical structures and high levels of interorganizational dependencies ( **1**) among several healthcare organizations. Still, today’s healthcare environment is governed by reaching increased levels of efficient delivery so as providers may manage the healthcare consumers in a cost-effective manner.

There are many sources of advantage but if they were to be grouped, they would be labeled the 5PS ( **2**): **Pace** refers to the timing and intensity of strategic action; **Position** is the projection of distinctive and desired images to the consumers; **Potential** is the access to superior capabilities and resources; **Performance** represents the superiority in actions and the implementation of strategy; **Power** is the outcome of effective organizational mass.

In ophthalmology services, the 5PS are reflected in the cultivation of referrals. As such, the goal of practicing an active referral management is the influence of patient flow. Referrers may be frequent, first time, local, discipline-specific, and even with high or low-income potential. The criteria used for obtaining positive referrals may focus on satisfaction with patient care, collegiality, and professional cooperation or discharge procedures.

Further, in order to strengthen the competitiveness in ophthalmology services, managers should invest in modern medical technology that may determine accurate diagnoses in due time and staff should follow training programs that concentrate on the improved individual skills in management and technology.

The key elements that would lead to a competitive advantage in ophthalmology services are the following: differentiation, innovation, consumer satisfaction, relevance of several investments and, the most important one, referrals.

**Assist. Prof. Consuela-Mădălina Gheorghe, PhD,** **Philologist, Authorized translator**
